# Achieving Our Moemoeā: Community-Led Food Security Strategy Development

**DOI:** 10.1177/15248399231177051

**Published:** 2023-05-26

**Authors:** Manawatū Food Action Network, Christina Severinsen, Angelique Reweti

**Affiliations:** 1 Environment Network Manawatū, Palmerston North, New Zealand; 2 Massey University, Palmerston North, New Zealand

**Keywords:** food security, community development, community-led, food resilience, collective impact, collective action, strategy, Whānau ora, community health promotion

## Abstract

The Manawatū Food Action Network (MFAN) is a collective of social service and environmental organizations and community stakeholders that work together to promote collaboration, education and awareness of issues surrounding food security, food resilience, and localization in the local community. In 2021, the 4412 neighborhood was identified as requiring urgent assistance, with approximately one third of residents experiencing food insecurity. The 4412 Kai Resilience Strategy was developed with the community to move from food insecurity to food resilience and sovereignty. Recognizing that food security is complex and based on multiple causes, six interwoven workstreams were identified to create a multifaceted, coordinated strategy. This includes education, food economy, community, food support, māra kai, and social enterprise. The strategy cultivates local ownership and commitment to change. It creates a broader constituency of support, balancing the urgent need to feed people today with the long-term need to change systems through step-change initiatives. Through this approach, communities can better make sustainable and meaningful changes to their lives and circumstances rather than relying on external resources.

## Assessment of Need

### Food Security in 4412

The 4412 neighborhoods of Palmerston North, Aotearoa New Zealand, are home to more than 25,000 people. 4412 is the local postcode aligning with the neighborhoods of Awapuni, Cloverlea, Highbury, Takaro, Westbrook, and West End in Palmerston North city. It is an area rich in diversity, hope, skills, and connections but is also one in which many households struggle to access adequate food, with approximately one third of residents facing food insecurity. In theory, there is currently sufficient available food for residents of 4412. Agricultural producers surround the area, yet social inequity and other challenges mean this abundance of food does not translate to food security for many whānau (extended family group) within the community. As a result of the COVID-19 pandemic, existing systemic vulnerabilities and inequities were further exacerbated, raising awareness of food insecurity and emphasizing the importance of community-led action to achieve a secure and resilient food system. Food security is described by Signal et al. (2012) as a “wicked problem,” highly complex and based on multiple causes.

Ora Konnect is a whānau ora, collective impact initiative that supports whānau to drive their health and well-being in the South-Western suburbs of Palmerston North. This group identified a challenge in how food availability within the 4412 community intersects with access and use, which cannot be addressed by a single intervention or one organization alone. In collaboration with Ora Konnect, the Manawatū Food Action Network (MFAN) and community partners identified the determinants of food insecurity as mental and physical health, housing insecurity, gang culture, addiction, income, employment and education, transport, information and communication networks, the policy and economic environment, family dysfunction, land alienation, and intergenerational trauma. For example, whānau told of the loss of intergenerational skills of growing, preserving, and cooking food. In addition, half of 4412 residents live in rental housing where tenancy agreements and housing instability are barriers to growing food at home. Nine free food providers currently serve 4412 to redistribute food; however, these community organizations lack sufficient resources to meet the total demand. This prompted the MFAN to support the community in developing a Kai Resilience Strategy.

### Description of Initiative

MFAN recognized that plans to increase food resilience and sovereignty must be whānau and community-led, adopting an iterative approach to strategy development to incorporate the community’s moemoeā (dreams), knowledge, skills, and experience of the food system in 4412. There are many existing community-based solutions to kai security and resilience, including the hard work of residents to earn incomes and support livelihoods, knowledge about growing and cooking food, sharing networks among neighbors, and the actions of service providers. In bringing together key stakeholders from within the community, such as mana whenua, whānau, community organizations and providers, MFAN facilitated opportunities for the community to share knowledge, deepen understanding, and plan collaboratively and creatively. This discovery process included meetings, a half-day workshop, interviews, community resource mapping, and additional consultation forms. The strategy development also included a situational analysis, analyzing publicly available data and a literature review. This resulted in the development of a community-driven strategy to support the 4412 community’s move from food insecurity to food sovereignty and resilience.

## Outcomes

As part of the 4412 Kai Resilience Strategy, existing networks will be strengthened, new networks will be founded, and communication channels will be developed that foster a sense of belonging and well-being for whānau and the wider community. The following definitions were created by the community and underpin the strategy:

**Food security:** Whānau have reliable access to a sufficient quantity of affordable, nutritious food.
**Food resilience:** Whānau are able to buffer and withstand food disturbances. Food resilience connects communities through mutual support.
**Food sovereignty:** Whānau have the resources to gather healthy and culturally appropriate food produced through ecologically sound and sustainable methods. Whānau are aware of their right to define their own food systems and are active agents in the production and procurement of their own kai. Food sovereignty has positive community outcomes through food sharing, connections, and belonging.

In recognition of the need for a multifaceted, coordinated strategy to support whānau in moving toward food security and self-determination, the 4412 Kai Resilience Strategy identifies six focus workstreams that flow and connect: education, food economy, community, food support, social enterprises, and māra kai. The spirit of each workstream is represented in the associated whakataukī^1^ (see Table 1).

**Table 1:** The 4412 Kai Resilience Strategy

Workstream	Whakataukī
Education	*Ko te manu e kai ana i te miro, nōna te ngahere; ko te manu e kai ana i te mātauranga, nōna te ao.* The forest belongs to the bird who feasts on the miro berry; the world belongs to the bird who feasts on knowledge.
	Whānau experiencing food insecurity expressed a desire to access more education and cultural wisdom. The strategy will develop and support education initiatives that enhance the community’s ability to create greater whānau food sovereignty.
Food economy	*Nāu te rourou, nāku te rourou, ka ora ai te iwi.* With my food basket and your food basket, the people will thrive.
	Moving our food system from the current commercial duopoly to a system that provides local sovereignty and reduces food waste. The strategy will contribute to food resilience by developing more opportunities for whānau to buy locally sourced, fresh, healthy food at an affordable price. The lobbying of government and industry to break down barriers, increase access to healthy food, and reduce dependency on the supermarket will have flow-on effects on product competitiveness and market diversity. Changes to the local food economy will give whānau more choices in their food-purchasing decision-making.
Community	*Mā pango, mā whero, ka oti te mahi.* The work will be done by everyone.
	The 4412 area is full of strong, diverse communities, ethnicities, and faith-based groups, rich in human resources but needing help with resourcing and funding. The strategy identifies and supports community projects that will help build strong communities and enhance the mana of locals.
Food support	*Aroha atu, aroha mai, tātou tātou e.* Love flows out, love flows back in, we are all one.
	Food support organizations in the area are experiencing increased demand for their services. The strategy supports free food providers in alleviating the immediate concerns of reducing hunger, providing holistic support, creating community connections, and contributing to environmental health through reducing waste and redistribution. Creating stronger connections between food suppliers, food banks, free food organizations, and whānau will ensure continuing support for whānau.
Māra kai	*Poipoia te kākano kia puawai.* Nurture the seed so it may grow and blossom.
	Growing one’s own food is economical. It also enhances people’s mana-enhancing, creates connections and gives people control over what they eat. By supporting people through community gardens, home gardening, urban farming, and social enterprise, whānau will be able to provide their own fresh, healthy produce and grow extra for their neighbors.
Social enterprise	*He aha te mea o te ao? He tangata, he tangata, he tangata. What is the most important thing in the world? It is people.*
	The strategy supports 4412 food business start-ups through networking, resource-brokering, and education. Supporting local food businesses will create employment, enhance place-making, and lead by example to foster motivation for further local development. Community-led food resilience projects will be supported to grow their efforts into social enterprises to create employment and make them financially sustainable. As global supply chains strain through world events, developing more robust, local supply chains is paramount. These actions will lead to higher quality food being consumed and enhance the mana of the community.

## Next Steps

### Achieving the Moemoeā

**Image 1:**
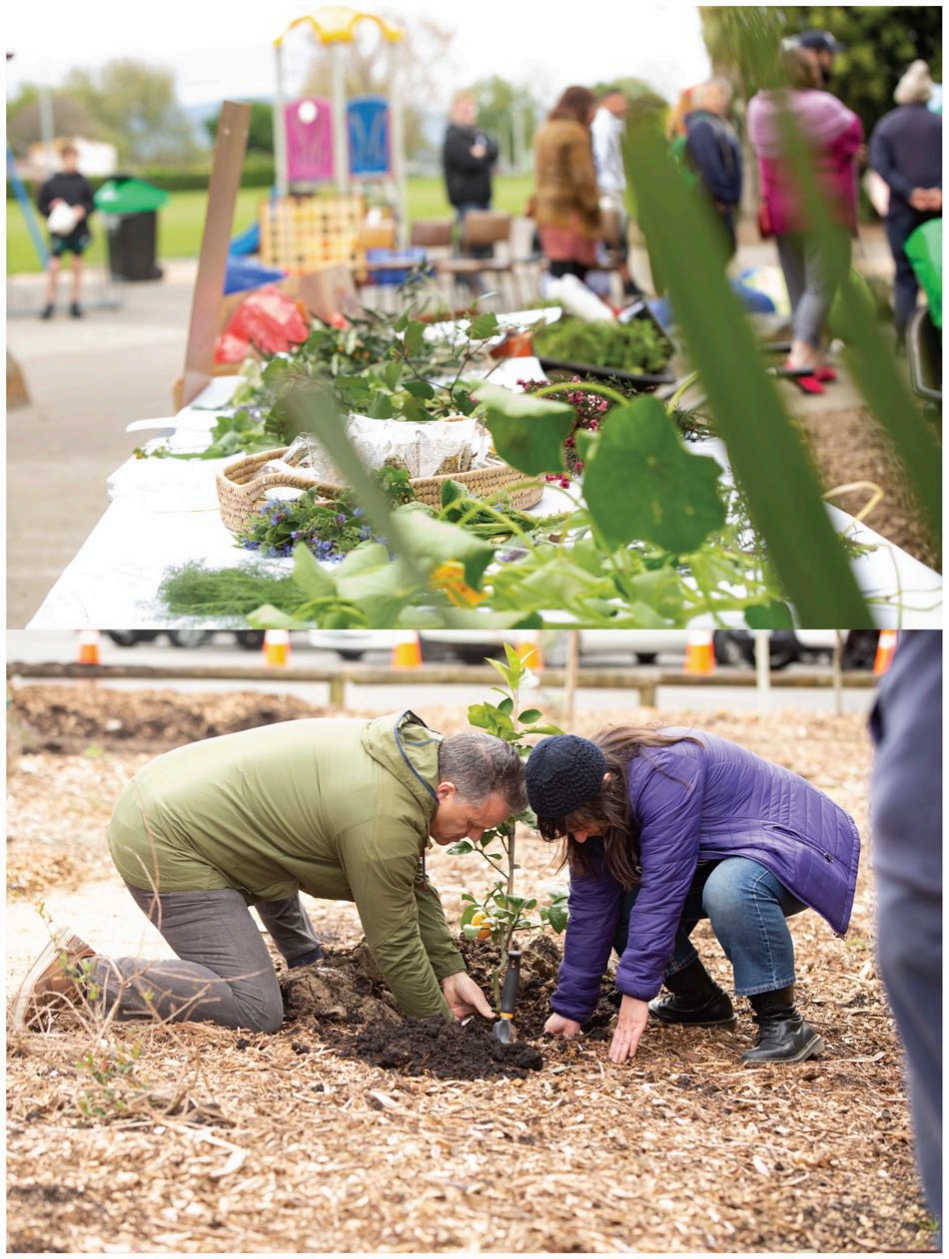
Community members come together for planting and sharing food

Within these streams, MFAN identified 114 projects that are either currently operating but need extra support, are planned, or are the moemoeā of the 4412 community. These projects range from macro-level food system change to meso-level community action to micro-level developing personal skills and providing urgent food support (see Image 1). Over 4 years, the strategy intends to build on whānau-led planning, increase food streams and sharing, and ultimately provide food sovereignty for the community. To understand where best to focus resources, projects will be prioritized into urgent, necessary, and desired. A calendar of community workshops and events regarding food resilience will be developed, and social enterprise will be fostered by supporting the establishment of multiple food start-ups.

### Implications for Practice

The 4412 Kai Resilience Strategy cultivates local ownership and commitment to change and creates a wider constituency of support, balancing the urgent need to feed people today with the long-term need to change systems through a series of step-change initiatives. Through this approach, communities can better make sustainable and meaningful changes to their lives and circumstances rather than relying on external resources.

## Notes

1. Whakataukī are Māori proverbs or significant sayings drawing on mātauranga Māori (Māori knowledge).
